# Sampling Real‐Time Atomic Dynamics in Metal Nanoparticles by Combining Experiments, Simulations, and Machine Learning

**DOI:** 10.1002/advs.202307261

**Published:** 2024-04-24

**Authors:** Matteo Cioni, Massimo Delle Piane, Daniela Polino, Daniele Rapetti, Martina Crippa, Ece Arslan Irmak, Sandra Van Aert, Sara Bals, Giovanni M. Pavan

**Affiliations:** ^1^ Department of Applied Science and Technology Politecnico di Torino Corso Duca degli Abruzzi 24 Torino 10129 Italy; ^2^ Department of Innovative Technologies University of Applied Sciences and Arts of Southern Switzerland Polo Universitario Lugano Campus Est, Via la Santa 1 Lugano‐Viganello 6962 Switzerland; ^3^ EMAT and NANOlab Center of Excellence University of Antwerp Groenenborgerlaan 171 Antwerp 2020 Belgium

**Keywords:** ADF‐STEM, atomic dynamics, metal nanoparticles, molecular dynamics simulations, unsupervised Machine Learning

## Abstract

Even at low temperatures, metal nanoparticles (NPs) possess atomic dynamics that are key for their properties but challenging to elucidate. Recent experimental advances allow obtaining atomic‐resolution snapshots of the NPs in realistic regimes, but data acquisition limitations hinder the experimental reconstruction of the atomic dynamics present within them. Molecular simulations have the advantage that these allow directly tracking the motion of atoms over time. However, these typically start from ideal/perfect NP structures and, suffering from sampling limits, provide results that are often dependent on the initial/putative structure and remain purely indicative. Here, by combining state‐of‐the‐art experimental and computational approaches, how it is possible to tackle the limitations of both approaches and resolve the atomistic dynamics present in metal NPs in realistic conditions is demonstrated. Annular dark‐field scanning transmission electron microscopy enables the acquisition of ten high‐resolution images of an Au NP at intervals of 0.6 s. These are used to reconstruct atomistic 3D models of the real NP used to run ten independent molecular dynamics simulations. Machine learning analyses of the simulation trajectories allow resolving the real‐time atomic dynamics present within the NP. This provides a robust combined experimental/computational approach to characterize the structural dynamics of metal NPs in realistic conditions.

## Graphical abstract

Experimental and computational techniques are bridged to unveil atomic dynamics in gold nanoparticles (NPs), using annular dark‐field scanning transmission electron microscopy and molecular dynamics simulations informed by machine learning. The approach provides unprecedented insights into the real‐time structural behaviors of NPs, merging state‐of‐the‐art techniques to accurately characterize their dynamics under realistic conditions.

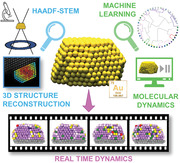

## 1 Introduction

Metallic nanoparticles (NPs), owing to their unique physicochemical characteristics, have garnered significant attention across diverse scientific and technological domains.^[^ ^1^, ^2^, ^3^, ^4^, ^5^ ^]^ Their magnetic, electrical, optical, chemical, and catalytic properties can be finely controlled by tuning their size, shape, and composition, leading to their broad applicability in various fields.^[^ ^6^, ^7^ ^]^ Specifically, gold (Au) NPs smaller than 3–5 nm exhibit elevated reactivity, making them suitable for use in a range of biomedical and catalytic applications.^[^ ^8^, ^9^, ^10^, ^11^ ^]^

The high surface mobility of Au NPs is a characteristic feature that plays a critical role in determining their unique properties.^[^ ^12^, ^13^, ^14^ ^]^ This is particularly important in small NPs, where the high surface‐to‐volume ratio results in a significant proportion of atoms residing on the NP surface, displaying greater mobility compared to those in the bulk even at fairly low temperature^[^ ^15^, ^16^, ^17^, ^18^ ^]^ The dynamic atomic rearrangements occurring on the NP surface significantly influence the optical, electronic, and catalytic properties.^[^ ^12^, ^19^, ^20^, ^21^, ^22^ ^]^ However, the high atomic mobility of NPs introduces substantial challenges for both experimental and theoretical investigations into NP structures. Given the highly dynamic nature of the atoms, capturing not just the static structure of NPs but also their real‐time atomic dynamics is crucial. This understanding is a key to fully grasp the behavior of Au NPs and effectively control their properties for different applications, thus the pressing demand for techniques capable of providing atom‐level insights into the dynamic behavior of NPs.

Despite the vast potential of Au NPs, a comprehensive understanding of their real‐time atomic dynamics under operational conditions remains elusive, limiting our ability to harness their full capabilities. Traditional experimental approaches typically lack sufficient resolution to track the dynamic behavior of individual atoms within these NPs.

Recent experimental advancements such as annular dark‐field scanning transmission electron microscopy (ADF‐STEM), have recently allowed reconstruction of the atomistic structure of NP from microscopy images taken at temperatures relevant to various applications.^[^ ^15^, ^23^, ^24^ ^]^ However, albeit ADF‐STEM offers a high (atomistic) resolution,^[^ ^24^ ^]^ a major limitation in this approach lies in the discrepancy between the frequency of the experimental image acquisition and the timescales that characterize the NPs dynamics. In particular, typical ADF‐STEM experimental setups can capture snapshots of the NP structure at intervals of ≈0.1–1 s.^[^ ^24^ ^]^ The real atomic dynamic present within them may nonetheless unfold at much shorter timescales, typically in the *ps* and *ns* scales^[^ ^13^ ^]^ Therefore, while experimental techniques such as ADF‐STEM allow for the collection of images and structures of NPs on a timescale relevant to experiments, the limited time resolution in data acquisition hinders the tracking of individual atomic movements within them. While techniques such as ultra‐fast electron diffraction (UED) excel in capturing atomic dynamics with enhanced temporal resolution, ranging from *fs* to *ps*,^[^ ^25^, ^26^, ^27^ ^]^ and high‐resolution transmission electron microscopy (HRTEM) extends this capability into the millisecond (ms) to second (s) range,^[^ ^28^, ^29^ ^]^ HAADF‐STEM stands out by providing indispensable 3D structural information.^[^ ^24^, ^30^ ^]^ Indeed, the integration of such method with molecular dynamics simulations creates a powerful workflow; this synergy leverages HAADF‐STEM's detailed spatial and 3D structural insights with the higher temporal resolution of MD simulations, offering a comprehensive approach to understanding the intricate behaviors and structures of nanoparticles

Computer simulations, namely atomistic molecular dynamic simulations that rely on reliable and accurate force fields, demonstrated to be remarkably useful for extracting data to understand the atomic dynamics within nanoparticles in relevant conditions.^[^ ^13^, ^31^, ^32^ ^]^ In particular, recently, the use of advanced structural and dynamical descriptors—e.g., smooth overlap of atomic positions (SOAP),^[^ ^33^ ^]^ local environments and neighbors shuffling (LENS),^[^ ^34^ ^]^ TimeSOAP^[^ ^35^ ^]^—allowed via machine learning to reconstruct the atomic environments populating the NPs in realistic conditions and the atomic dynamics present within them; it should be noted, however, that such analyses are obtained from MD trajectories acquired from simulations that start from ideal nanoparticle structures, which may differ from the structure typical of the same nanoparticles under experimentally relevant conditions. This makes it difficult to guarantee that the extracted data provide a reliable reconstruction of the equilibrium dynamics of these NPs since one of the main limitations of classical MD simulations lies in the sampling and in the risk of entrapment in local energy minima.

In this work, we demonstrate the potential of combining state‐of‐the‐art experimental and computational approaches, to overcome the limitations of each method. Particularly, simulations, by enabling the observation of rapid atomic movements within NPs over suitable time scales, serve as a bridge between static imaging and the dynamic behavior of NPs, thus addressing the challenges associated with time scale decoupling. This integrative method not only addresses the individual challenges posed by experimental and computational techniques but also offers a comprehensive analysis of NP dynamics that is closely reflective of real‐world conditions, thereby deepening our understanding of NP behavior. Our method begins with the acquisition of high‐resolution ADF‐STEM images, which enable precise 3D reconstructions of the NP (through an iterative local minima search algorithm^[^ ^24^ ^]^). Specifically, we used 10 NP configurations each captured at 0.6 s intervals, during 6 sof ADF‐STEM experimental data acquisition. These experimentally reconstructed structures provided a detailed view of the actual atomic arrangements in supported Au NPs, capturing complexities and deviations from idealized structures, and aligning our simulations with real‐world behaviors of Au NPs. These reconstructed NP structures serve as starting points for as many MD simulations, which then allow us to capture the real‐time dynamics of NP. This approach enables us to run atomistic MD simulations relying on experimental‐level sampling, studying the dynamics of Au NPs at different T and obtaining results in line with the experimental observations. Our analysis demonstrates that the dynamics we can reconstruct from MD simulations represent an equilibrium ensemble. Our computational strategy leverages advanced descriptors like SOAP and LENS, integrated with data‐analysis workflows, to accurately reconstruct NPs in realistic conditions, marking a significant breakthrough in modeling dynamic atomic environments. The level of insight that can be obtained with this combined experimental/computational approach provides us with a versatile tool to understand the real atomic‐scale dynamics of metal NPs and to link this to their macroscopic properties.

## 2 Results

### 2.1 Outline of the Combined Experimental‐Computational Approach

**Figure 1:**
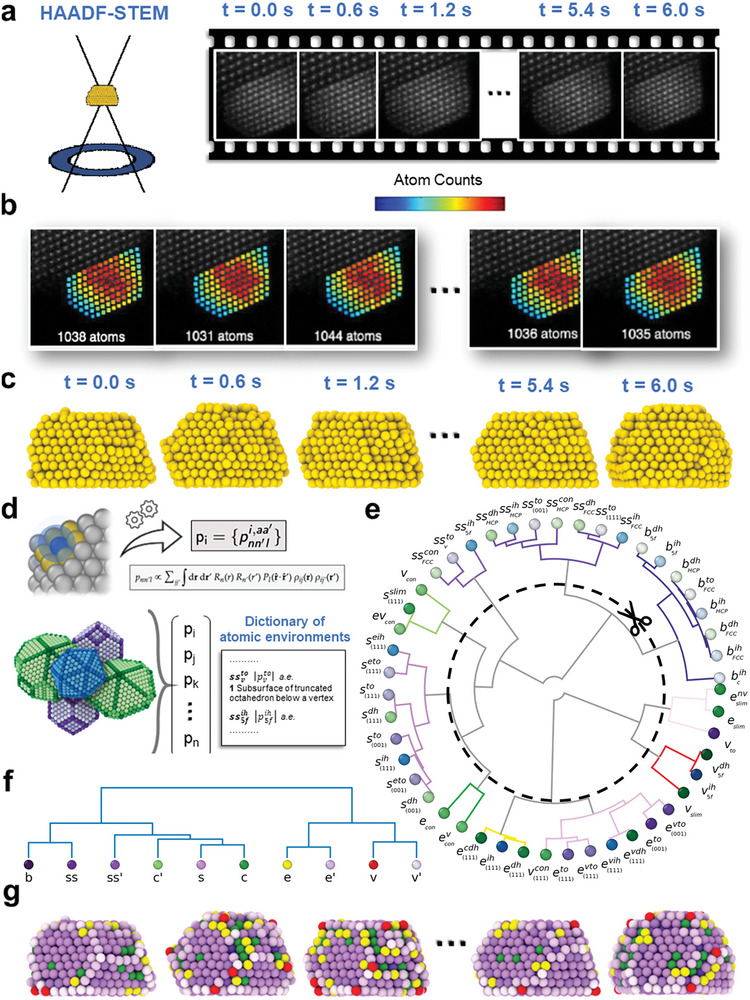
Stepwise process of applying the SOAP analysis to the experimental structures of Au (NPs). a) Left: Schematic representation of the High‐Angle Annular Dark Field Scanning Transmission Electron Microscopy (HAADF‐STEM). Right: Ten consecutive ADF‐STEM frames of observed Au NPs at 673 K. b) Atom‐counting maps corresponding to the ten frames from (a), with the color indicating the atom count per column. c) Final reconstructed 3D structures of the observed NPs. d) Top: Each atom in an Au NP (depicted in blue) is assigned a SOAP vector, with the cutoff radius shown as a transparent sphere (*r*_*cut*_ = ≈4.48 Å, corresponding to 110% of the Au FCC lattice parameter). Bottom: Construction of a SOAP dictionary of atomic environments (AEs) using icosahedral (blue), decahedral (green), and truncated‐octahedral (purple) Au NPs, which also includes larger NPs for comprehensive AE representation. e) A global dendrogram connecting AEs from different NPs. This dendrogram visualizes hierarchical clustering of AEs based on SOAP distances, with branches connecting the different clusters. By cutting at a SOAP distance threshold of 0.08 we form a coarse‐grained dictionary, reported in (f). g) Final 3D structures, with atoms colored according to the SOAP classifications from the AEs dictionary.

A recent breakthrough in our team involves a novel approach that combines atom‐counting and iterative local minima search algorithms, while also considering temperature effects, to reconstruct the 3D structure of supported NPs using single‐view 2D ADF‐STEM images (**Figure** **1**a).^[^ ^15^, ^23^ ^]^ This technique was applied in the demonstrative case study reported herein to Au NPs supported on CeO_2_ at a temperature of 673 K. The methodology begins with ADF STEM imaging, essential for attaining atomic‐level details of nanoparticles. This step is enhanced by advanced atom‐counting techniques employing statistical parameter estimation, which precisely determines the composition and density of the NP. The data obtained from these techniques lay the groundwork for the next phase, involving the integration of these findings into MD simulations to create a preliminary 3D model of the NP.^[^ ^24^ ^]^

Further refinement of this initial model is achieved through a local minima search algorithm which adjusts atomic positions within the model and thoroughly evaluates the system's energy landscape. These careful adjustments are key in approximating the NP's real structure.

An innovative element of this approach is the incorporation of molecular dynamics structural relaxation at an experimental temperature of 673 K. This step is vital to ensure that the reconstructed models are not only theoretically accurate but also realistically represent the nanoparticles' behavior under specific, experimentally relevant temperature conditions;^[^ ^9^, ^36^, ^37^, ^38^, ^39^ ^]^ indeed, aligning the models with real‐world scenarios are crucial for their practical applicability.

Validation of the reconstructed 3D structures is conducted through an extensive comparison with experimental data. This validation focuses on the precision of atom positions and the overall morphology of the nanoparticles, ensuring that the reconstructed models are not only theoretically sound but also congruent with observed experimental behaviors. This step is crucial in confirming the practical viability and accuracy of the reconstructed models.

Readers interested in a deeper dive into the specificities of this 3D reconstruction process are directed to the dedicated subsection within the Methods section of this paper. Additionally, for an expansive overview and detailed methodological insights, reference paper^[^ ^24^ ^]^ offers extensive information.

In particular, this approach was used as a first step to generate a series of atom‐counting maps from 10 snapshots taken every 0.6 s along a 6 s of ADF‐STEM sampling (Figure 1a). In Figure 1b we present snapshots of the obtained NPs, color‐coded based on a scheme that corresponds to the number of atoms in each atomic column. As previously mentioned, the reconstructed 3D structures of the NPs correspond to 10 frames, captured over a total observation time of 6 s using ADF‐STEM^[^ ^24^ ^]^ (Figure 1c). Although the time intervals between the reconstructed NP structures are relatively long (0.6 s), they significantly contribute to our comprehension of atomic surface dynamics in environments similar to practical applications.^[^ ^9^, ^36^, ^37^, ^39^ ^]^ This provides a profound insight into the dynamic activities on NP surfaces, reflecting conditions encountered in real‐world experimental settings. To extract information on the atomic environments (AEs) emerging on the NP during the experimental data acquisition, we employed a recently designed approach^[^ ^13^ ^]^ based on SOAP power spectra^[^ ^33^ ^]^ (Figure 1d), with the purpose of analyzing and better interpreting what happens in the atomic structure of these real NP experimental snapshots. As a first step, we calculated the SOAP spectrum of each atom for all the snapshots (see Experimental Section for details). The SOAP power spectrum provides a comprehensive representation of the atomic environment by combining radial basis functions and spherical harmonics, effectively capturing both the local structural details and global symmetry features.^[^ ^33^, ^40^ ^]^ This descriptor thus provides deep insight into how the neighbor atoms are arranged in the space in the local environment surrounding each atom in the NP (*i.e* within a cutoff radius).^[^ ^13^, ^41^, ^42^, ^43^ ^]^ Complete details on the SOAP analysis are available in the Experimental Section.

The extracted SOAP spectra (2 × 10^6^ for each of the 10 frames) of the Au atoms have been then classified based on a general AE dictionary of Au NP SOAP AEs, that we recently developed.^[^ ^13^ ^]^ Such comprehensive AE dictionary includes 47 SOAP spectra of all the AEs present in ideal Au NPs (at 0 K) of various sizes and morphologies at 0 K (Figure 1d). This provides us with an essential tool for monitoring the AEs that are present in the real NP under experimental conditions and classifying them based on their similarity to the AEs contained in the dictionary. Figure 1e displays the 47 AEs defined in our SOAP environments dictionary, presented in a circular dendrogram arranged according to their similarity based on SOAP fingerprints. The dotted inner circle indicates that truncating the dendrogram, we consider only SOAP distance larger than 0.8 as relevant^[^ ^13^ ^]^ (an excessive resolution increases the noise and would emphasize irrelevant differences). This choice simplifies the dictionary from 47 to 11 without sacrificing significant variations^[^ ^13^ ^]^ (Figure 1f). In the adopted color scheme of the dendrogram of Figure 1f, the colors belonging to the purple palette refer to native AEs typical of ideal truncated octahedral NPs, while all the other colors identify AEs that are more similar to AEs proper of other ideal NPs' morphologies (e.g. icosahedral, decahedral, etc.)

Leveraging such “coarse‐grained” dictionary, we colored the atoms in the ten experimentally reconstructed NP structures based on the similarity of their SOAP atomic environments to those within our SOAP dictionary (Figure 1g). This first analysis reveals the dynamic nature of the atomic environments within the NPs in experimental conditions over the 6 s data acquisition, during which the atoms of this octahedral Au NP moves, and non‐native AEs (colored in yellow, green, and red) emerges on the NP surface. Far from being static, these NP structures show remarkable variability driven by thermal effects. The positions of vertices and edges within the NP displayed substantial shifts, underscoring the ongoing structural transformations. Our method thus provides a unique view of the atomic‐level dynamics of NP, using Machine Learning (ML) analysis to illuminate structural evolution from experimental static frames.

**Figure 2:**
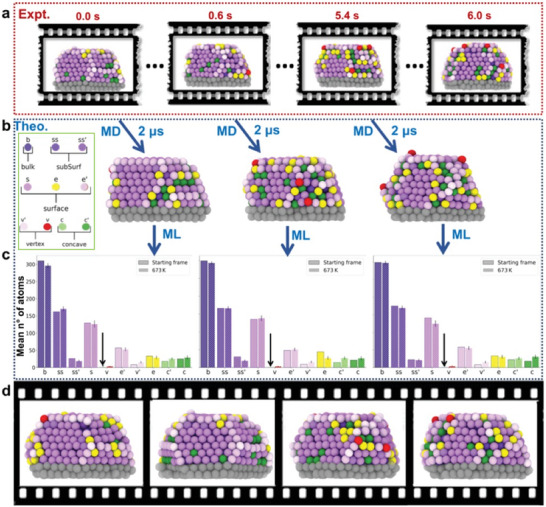
Schematic representation of the combined approach for capturing atomic‐scale dynamics in Au NPs. a) Experimental frames showing the time resolution of 0.6 s, highlighting the temporal gaps between the snapshots. b) Final frames after 2 µs MD simulations, with atoms colored according to the dictionary developed from the SOAP spectra analysis, effectively filling the temporal gaps between experimental snapshots. c) Histograms displaying the average count of atoms associated with each atomic environment, providing a measure of system stability. Standard deviations as vertical black lines. d) Conceptual representation of the full 6 s movie reconstruction, with gaps filled by MD simulations.

Such atomic‐level ML analysis underlines that understanding the atomic dynamics present in these NPs is key to comprehend their properties in experimentally‐relevant conditions. While these experimental snapshots of the NP structures provide initial important evidence, they are spaced by time intervals of 0.6 s (**Figure** **2**a). This is a substantial time gap, especially considering that atomic dynamics typically unfold on much faster timescales, such as pico‐ and nano‐seconds. This temporal mismatch makes it impossible to reconstruct the atomic dynamics directly from such ADF‐STEM reconstructed snapshots, as, e.g., it is not possible to attribute an identity to the individual atoms nor to monitor their movements from one snapshot to the subsequent one.

To address this issue, we utilized the high spatiotemporal sampling resolution provided by atomistic molecular dynamics (MD) simulations, which allow for more detailed tracking of atomic movements.

### 2.2 Combining Atomistic‐Scale MD Simulations and Experimental Level Sampling

We initiated ten MD simulations using the ten experimentally reconstructed configurations depicted in Figure 2a, with each starting structure containing a varying number of atoms ranging from 1031 to 1044. These simulations were conducted at temperatures of 300 and 673 K (consistent with experimental conditions^[^ ^9^, ^36^, ^37^, ^39^ ^]^), enabling us to obtain trajectories from which it is possible to track the movements of the individual atoms in the NP and between the AEs that emerge within them; in these simulations, we employed the SMATB potential,^[^ ^44^, ^45^, ^46^ ^]^ which has been demonstrated to accurately describe the dynamics of gold NPs.^[^ ^13^ ^]^ It is important to note that we did not include the substrate in our simulations. For more detailed information on our simulation methodology, please refer to the Experimental Section. Each MD simulation lasts for 2 µs, and we collected 1000 frames (every 1 ns in MD) from the final 1 µs of the trajectory. During this period, we computed SOAP spectra for all the atoms in the NP. It's important to mention that the chosen time window for our analysis ensures that any observed communication or exchange among the AEs pertains to processes occurring on the nanosecond timescale or slower. This effectively reduces the likelihood that the AE exchanges are influenced by thermal vibrations. This led to ≈10^6^ SOAP spectra for each MD, comprehensive for 10^7^ SOAP spectra for all the 10 MD simulations. In this SOAP analysis, we utilized a cutoff radius of 4.48 Å, corresponding to 110% of the lattice pair distance of gold, which is included in the calculation up to the first two neighbors. The selection of the cutoff is a critical decision,^[^ ^47^ ^]^ and for this reason, this specific value was chosen to achieve a balance between computational efficiency and the fidelity of information retained, ensuring a thorough and accurate representation in our analysis.^[^ ^13^ ^]^ Differently from the previous analysis on the static experimental frames, these MD simulations permit us to track the SOAP AEs to which each atom of the NP belongs to over time. This allows us, e.g. to quantify the propensity of each atom to remain in a certain AE or to undergo a transition to a different one at every δt (1 ns). By delving deeper into the dynamics of these atomic environments, we were able to quantify the NP's stability and analyze the dynamics of exchange between its various constitutive AEs. We generated histograms from the final 1 µs of the MD simulations, which provide an average atom count associated with the population of each AE (Figure 2c). Comparing the histograms obtained, e.g., at 673 K versus those of the starting NP configurations, provides information on the NP stability and it is interesting to note how the AEs histograms calculated from the MD at 673 K do not deviate much from those obtained from the experimental structures, despite the considerable dynamics observed along the MD.

Figure 2b,c shows that during the various MD runs, dynamic atomic rearrangements can be observed; vertical arrows in the histograms indicate AEs that are not present in the starting frame, but that may emerge with temperature: e.g. red “v” AE. Furthermore, Figure 2b,c (left to right) shows that, while some variability between the MD runs can be expected, the histograms do not change much in the various systems configurations. This implies that these MD simulations offer a detailed view of the atomic dynamics within these NPs. By observing them at an atomistic resolution over microsecond‐long time windows, we can track their equilibrium trajectories under conditions that are relevant to real‐world experiments. This allows concatenating the various 1µs‐long analyzed MD trajectories, obtaining 10 µs of sampling of the equilibrium MD of the Au NP.

We repeated the same analysis, as a control case, by running the 10 MD simulations at a lower temperature (300 K). In this case, the analysis shows, as expected, less dynamic activity in the NPs compared to 673 K. However, the overall conclusions remain the same (complete data at 300K are provided in the supporting information‐ Figure S1, Supporting Information).

**Figure 3:**
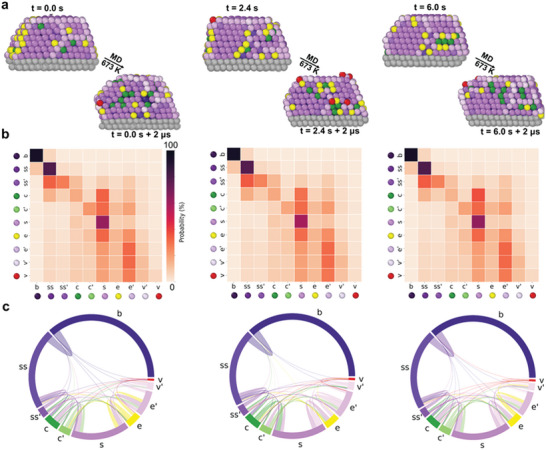
Quantitative analysis of atomic transitions and stability in reconstructed Au NPs at different temperatures. a) Initial structures at time points 0.0, 2.4, and 6.0 s, and the corresponding final structures post‐MD simulations at 673 K, color‐coded according to the SOAP dictionary. b) Normalized transition matrices demonstrating the probability of atoms remaining in a given AE*i* (*p*_*ii*_) or transitioning to a different AE*j* (*p*_*i* → *j*_) within a time interval of δ*t* = 1 ns. c) Chord diagrams showing the dynamic exchanges between environments; the size of each arc section is proportional to the population of each AE, while the width of the chords represents the intensity of exchanges between them.

The next step involves a statistical analysis of the SOAP data, which allows us to quantify the dynamics of the NP. **Figure** **3**a illustrates the initial structures taken at 0, 2.4, and 6 s, and the corresponding structures obtained after 2µs of MD at 673 K (color code according to the dictionary of Figure 1).

The NP surface maintains its structural integrity throughout the simulation, suggesting that the truncated octahedral structure remains relatively stable overall, despite the high atomic mobility. To characterize the intricate atomic dynamics present in the NPs, we calculated the transition probabilities for atoms between these AEs.

The transition matrices in Figure 3b indicate the probability of an atom, with a specific AE at time *t*_*i*_, remaining in the same AE (diagonal entries) or transitioning into a different AE (off‐diagonal entries) within the analysis time interval (δ*t*).

At 673 K, all cells in the transition matrix are colored, indicating non‐zero transition probabilities across all AEs within the NP. This observation indicates a marked increase in atomic mobility and interchange between various NP regions at higher temperatures.^[^ ^13^, ^41^, ^42^, ^48^ ^]^

A visual representation of these dynamic exchanges is well rendered by the use of chord diagrams of Figure 3c, where the size of the arc sections is proportional to the population of the various AE, while the width of the chords corresponds to the intensity of atomic exchanges between them. At 673 K, these analyses show high communication and dynamic exchange in the NP. This does not pertain to surface AEs only but this atomic exchange can also be observed between the innermost NP bulk (*b*) and the least coordinated surface AEs, e.g., vertexes AEs (*v* and *v*′), or between sub‐surface and surface AEs (*ss*→*v* and *ss*→*e'*). On the other hand, at 300 K, dynamic exchanges are predominantly observed among AEs with similar coordination numbers, such as *v*→*v'* and *b*→*ss*, indicating limited atomic mobility and constrained transitions. Despite the striking internal atomic dynamics observed at 673 K, in such conditions, the NP still preserves its truncated octahedral structure (Figure 3); it is interesting to note that these analyses performed on the MD trajectories starting from the experimental configurations captured at 0, 0.6,…,6.0 s provide very similar results (Figure 3b,c left‐to‐right). This demonstrates that these MD provide reliable pictures of the internal atomic dynamics present in these NPs in equilibrium regimes and in experimental relevant conditions. Furthermore, this also allows us to concatenate all data in a unique dataset, useful to improve the statistical confidence of our analysis.

### 2.3 Realistic Atomic Dynamics in Au NPs in Experimentally‐Relevant Conditions

**Figure 4:**
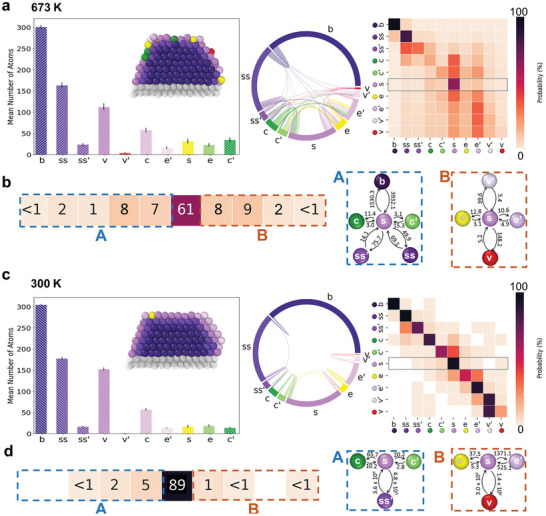
Detailed analysis of atomic transitions and mobility in Au NPs across the experimental time‐scale. a) Equilibrium representation of atomic environments at 673 K, featuring histograms similar to Figure 3, confirming AE distribution. The inset shows the equilibrium 3D structure of the NP, cut to show the interior. In the middle and right panels, chord diagrams and transition matrices encapsulate the aggregated fluxes and transition probabilities for the combined trajectories, representing atomic behaviors over the full experimental time. b) Focused examination of specific transition probabilities from the surface (*s*) AEs at 673 K, demonstrating the likelihood of atoms on the NP's flat faces transitioning to other AEs. The rightmost panels display the characteristic times (in ns) of transitions related to surface AEs. Panels (c) and (d) report the same results, but at 300K.

Merging together the results obtained from the ten (independent) MD trajectories, we could obtain an average and comprehensive picture of the dynamics of Ceria‐supported Au NPs. **Figure** **4** averages the data derived from our MD trajectories providing meaningful insights into the atomic ensemble's dynamics and transitions present in these NPs over the entire experimental sampling timescale. Figure 4a,c provides an equilibrium representation of the AEs dynamics at 300 and 673K respectively. The average histograms, similar to those in Figure 3, confirm our earlier observations of the AE distribution, showing that the NP's dynamics at thermal equilibrium maintain similar characteristics over the full experimental time‐scale. The chord diagrams and transition matrices, displayed in Figure 4a (middle and right) and Figure 4c (middle and right), capture at atomistic resolution the average AEs transition probabilities that characterize such NP in experimental conditions and in relevant observation timescales, obtained from the ten individual 1µ*s* MD windows. These average diagrams reveal similar patterns to the ones observed in (Figure 3), taken along 6s of global experimental samplings, providing a statistically robust perspective of the equilibrium atomic structure and dynamics of these NPs. The details of panels Figure 4b,d show how selecting one specific AE (e.g. *s*), from the matrices one can obtain a specific transition probability of the dynamic interconnections with the other ones in the NP. From transition probabilities, between AEi and AEj (*p*_*i* → *j*_) one can obtain information on the average lifetimes of different AEs within the NP and on the average transition rates for the atomic exchange between them. The off‐diagonal elements of the transition matrix, denoted as *p*_*i* → *j*_, provide data on the probability for transitioning from AE*i* to AE*j* over the sampling time interval (δ*t*), which we preset at 1 ns for our investigation. This choice represents the best compromise between capturing significant atomic AE exchanges and managing computational costs. This period effectively balances resolution and computational efficiency, ensuring that observed AE exchanges reflect sustained, dynamic processes within the NPs. This time‐frame minimizes the influence of transient thermal fluctuations, focusing on the more substantial changes we aim to observe, and aligns with our goal of accurately depicting meaningful dynamics in nanoparticle behavior.

We observe a high mobility of atoms within this AE at 673 K, where transitions are observed to/from all other AEs, including the innermost bulk AE. Specifically, at 673 K, surface atoms have a probability of ≈61% of remaining in AE *s* during the 1‐ns sampling interval.

This probability increases to ≈91% at 300K. However, even at this lower temperature, transitions from the surface (*s*) to the concave AEs (*c*, *c*′) and edges (*e*) can still be observed with probabilities significantly above ≈1% (in *dt* = 1*ns*).

By dividing *p*_*i* → *j*_ by *dt*, one can estimate the transition rate (*k*_*i* → *j*_), and consequently calculate the characteristic timescale (τ_*i* → *j*_) expected for each transition (the reciprocal of *k*_*i* → *j*_). The right parts of Figure 4b and Figure 4d show the characteristic timescale of transitions involving surface AEs (*s*). Comparing the data extracted at 673 and 300 K, the transition times diminish substantially at higher temperatures, exemplified by the characteristic timescales for *s*→*c* transition shrinking from ≈66 ns at 300K to ≈11 ns at 673K. Such different dynamics as a function of temperature, are even more pronounced for transitions between the inner AEs; the interior of the NP is almost static at 300 K, e.g., the *s*→*ss'* (i.e., surface to subsurface) transition time drops from ≈10^4^ ns at 300K to ≈14 ns at 673 K, a diminution of three orders of magnitude. These analyses demonstrate how such a combined experimental/computational approach allows achieving a resolution of the *ps* scale, to reconstruct the atomic dynamics present over a real 6 s experimental time window. Such level of detail provides crucial insights, such as, e.g., how long an AE exists in realistic conditions, which is key to understanding the surface properties of these NPs.

## 3 Rare Local Transitions on the NP Surface in Experimental Conditions

While the equilibrium and average dynamic picture discussed thus far are useful, they could mask significant local fluctuations, key for the NP properties. In particular, such analyses based on pattern recognition of the statistically dominant AEs may lose information, in particular, on fluctuations/transitions that may sparsely occur along the MD trajectories and that have negligible statistical weight. To address this issue, we have completed our previous analysis using a different abstract descriptor called “Local Environments and Neighbors Shuffling” (LENS).^[^ ^34^ ^]^ LENS allows detecting and tracking rare local fluctuations, by monitoring how much every Au atom in the NP changes neighbor individual atoms identities (IDs) every 1 ns along the MD trajectories, which are typically overlooked in pattern recognition structural (e.g., SOAP) based analyses.

**Figure 5:**
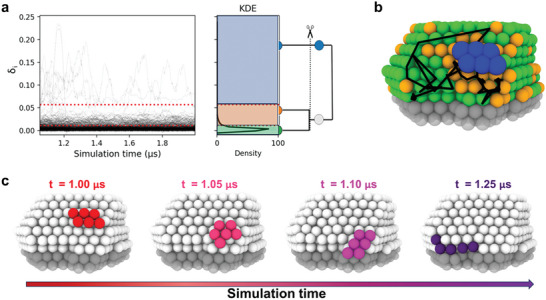
Exploring local fluctuations and dynamic domains within reconstructed Au NPs. a) Left: Time‐series of LENS signals (δ_*i*_(*t*)), Kernel Density Estimate (KDE) of the LENS distribution,^[^ ^34^ ^]^ and interconnection dendrogram for a 1‐microsecond window of our Au NP MD at 300K. The KDE provides an overview of the LENS distribution, while the dendrogram illustrates the interconnections between dynamic domains. b) An example of a local fluctuation, where a small group of Au atoms exhibits rapid diffusion on the NP surface. Atoms are colored according to the LENS cluster represented in the KDE plot. The corresponding movements during the MD simulation on the underlying NP surface are highlighted by black trajectory lines. This dynamic behavior of a localized group of atoms adds complexity to the overall NP behavior, with potential implications for system properties and reactivity. c) Four snapshots at different times (indicated above) illustrate the movement of the aforementioned group of dynamic atoms. The color scheme used is relative to the simulation time, as represented by the arrow below.

**Figure** 5a illustrates the time‐series of LENS signals, denoted as δ_*i*_(*t*), obtained from one of the MD trajectories at 300K. These signals were computed starting from the experimentally reconstructed structure obtained at 2.4 s. Additionally, the figure includes the Kernel Density Estimate (KDE) of the LENS distribution and the interconnection dendrogram, covering the final 1µs of our AuNP MD simulation at 300K.

Atoms exhibiting elevated δ_*i*_(*t*) values are suggestive of pronounced dynamism within their atomic environments. These elevated δ_*i*_(*t*) values are indicative of considerable variations in the number and identities of neighboring atoms, signifying notable changes in the local neighborhood of each atom. Consequently, atoms characterized by persistently elevated δ_*i*_(*t*) values are systematically classified into the more dynamically active regions. This criterion underpins our methodological approach in differentiating between various dynamical states of the atoms, with the most dynamically active clusters comprising those atoms exhibiting the highest values of δ_*i*_(*t*).

More precisely, by applying K‐means clustering^[^ ^49^ ^]^ to the LENS data, we identify 3 distinct environments, represented in the right panel of Figure 5a, characterized by different LENS signals (local dynamics), enabling the construction of an associated dendrogram illustrating their adjacency.

This detailed dynamic analysis complements our equilibrium SOAP analysis, pinpointing localized dynamic areas amidst a backdrop of relative stability, and underscoring the complexity of NP behavior. Indeed, in line with the equilibrium SOAP analysis, the majority of NP atoms remain within a relatively “static” region, visualized by the green domains. However, a sparse subset of atoms participates in local transitions and diffusion, leading to the emergence of localized dynamic regions shown in blue (*cf*. Movie S1, Supporting Information). Figure 5b provides an illustrative example of these local fluctuations, where a few Au atoms demonstrate rapid movements on the NP surface. Such infrequent behaviors, which may not be as evident in other analyses like those in Figure 4, are effectively detected by LENS, providing a deeper insight into the microscopic and macroscopic features characterizing the atomic dynamics of these metal NPs under experimentally‐relevant conditions.

## 4 Conclusion

In this work, we presented a combined experimental/computational approach that allows us to characterize the (complex) atomic dynamics present in Au NPs in realistic relevant conditions. Leveraging high‐resolution ADF‐STEM microscopy, we demonstrate the potential of this approach by obtaining ten images of a real Au NP every 0.6 s along a total of 6 s of experimental data acquisition. These images are then used to reconstruct as many atomic precise 3D structures of the Au NP, which are then used as a starting point for ten microseconds‐long independent MD simulations. By integrating advanced descriptors of atomic environments with machine learning, we can track the atomic‐scale rearrangements of the individual atoms on the NP over time, combining in this way the high spatiotemporal resolution of fully atomistic MD and the advantage of starting from multiple (time‐decorrelated – experimental dt = 0.6 s) NP structures. This allows us to minimize the typical sampling limitations of MD simulations starting, e.g., from initially perfect (ideal) NP structures.

MD simulations starting from NP structures taken every 0.6 s along a multi‐second experimental data acquisition show consistent and conserved atomic dynamics within the NP. This is true both at 673 K (same temperature as during the data acquisition) and at 300 K (see Figure 2, Figure 3 and Figure 4). Although the simulations at 673 K show pronounced atomic dynamics, the nanoparticle maintains its octahedral structure. This stability, observed under experimental conditions, illustrates that the dynamics in our simulations align with the equilibrium behavior expected in practical environments where nanoparticles are actively employed.^[^ ^9^, ^36^, ^37^, ^39^ ^]^ This means that such an approach allows us to reconstruct in a reliable—notably, with a spatial resolution of the Å, and the time resolution of the picosecond—way the structural dynamics that such metal NPs have when observed along an experimentally‐relevant seconds time‐window.^[^ ^24^ ^]^

The ML approach used herein to analyze the MD trajectories allows us to identify and classify all the AEs that emerge within the Au NP in realistic conditions, and to quantify, e.g., their lifetimes, transitions, etc. The dynamics reconstructed via SOAP and LENS data identify dominant average dynamic behaviors as well as sparse concerted movements, such as the “terrace sliding” on the NP face shown in Figure 5 that, being rare events, are typically difficult to capture with conventional pattern recognition approaches. On the one hand, such insights provide a new, exquisitely dynamic (qualitative) view of these atomic NPs (see Movie S1, Supporting Information). At the same time, the analyses of these MD simulations provide a useful approach to interpret and rationalize, in the future, the properties of such NPs in experimentally relevant conditions (e.g., their reactivity, stability, etc.).

We believe that the comprehensive understanding of the internal atomic dynamics that it is possible to attain for metal NPs with such a combined experimental/computational approach will offer a fundamental tool for rational control of NP properties. On a first simpler step, for example, it provides a direct way to understand the effect of the environmental conditions on the behavior expected from these NPs. Increasing with the complexity, it will also pave the way toward a better understanding of the properties of such metal systems, e.g., under the exposure to various stimuli or revealing, for example, the effect of the interaction with different entities (e.g., reactants, molecules, etc.) on the structural dynamics of the NP and, vice versa, the effect of the dynamics of the AEs that populate the NPs on the NPs' properties (e.g., interactions, reactivity, etc).

In summary, our work not only advances the current state of the art in studying metal NPs but also highlights the remarkable stability of these systems under realistic conditions. By integrating experimental data and computational simulations, we bridge the gap between theory and practice, offering valuable insights into the dynamic behavior of metal NPs in real‐world scenarios, with implications for catalysis and materials science. We are confident that our approach will serve as a foundation for further advancements in NP research, enabling precise control and optimization of NP properties for various applications.

## 5 Experimental Section

The NPs were stimulated using SMATB^[^ ^44^, ^45^, ^46^ ^]^ potential, available in LAMMPS,^[^ ^50^, ^51^ ^]^ acknowledged for its specific application to gold nanoparticles despite known limitations, such as underestimation of the melting temperature.^[^ ^52^ ^]^The approach was validated through extensive comparison with experimental data, emphasizing atom position precision and nanoparticle morphology to ensure theoretical and practical congruence of the models. The NP models were initially minimized using the built‐in command in LAMMPS (set up with etol = 10^−6^ ftol = 10^−8^, maxiter = 1000 and maxeval = 10000), then a small thermalization of 20 000 MD steps was performed with the timestep set to 1 fs on the NP with the velocities initialized to the desired temperature and with the thermostat with the same settings of the main simulation. Ten reconstructed Au NPs^[^ ^24^ ^]^ were stimulated at temperatures of 300 and 673 K. The number of atoms in each configuration, starting from the first structure to the tenth, are as follows: 1038, 1031, 1044, 1044, 1047, 1037, 1042, 1030, 1036, and 1035 All MD simulations were conducted in the canonical ensemble using the LAMMPS's Langevin thermostat, using a timestep of 5 fs, and a damping parameter for the Langevin thermostat set to 100 ps. Each NP system was stimulated for a total of 2 µs of MD. During the simulations, all NP systems reached a steady state in the MD regime (equilibrium). All the analyses were thus conducted on 1000 frames taken every 1 ns along the last 1 µ*s* of each MD simulation, during which the populations of all detected AEs were plateaued. It is important to note that in the simulations, the ceria (CeO_2_) substrate on which the gold nanoparticles were often supported was not explicitly included.^[^ ^38^, ^53^, ^54^, ^55^ ^]^ Instead, to mimic the effect of the substrate on the nanoparticles, a potential was applied to the last layers of the nanoparticle. This approach was chosen as the primary interest was in understanding the intrinsic dynamics of the gold nanoparticles themselves, rather than the interactions between the nanoparticles and the ceria substrate. By focusing on the gold nanoparticles and employing the SMATB potential, which had been shown to accurately describe the dynamic behavior of these systems,^[^ ^13^ ^]^ under the conditions of interest, the properties and behavior of the nanoparticles could be more easily investigated. A key observation from the study was that the structure obtained after MD simulations closely resembles the structure reconstructed experimentally. This similarity indicates that the potential applied successfully reproduced the impact of the substrate on the structure of the nanoparticle. This decision to omit the explicit simulation of the substrate allowed to concentrate the computational resources and analysis on the aspects most critical to the aim

### SOAP Analysis

SOAP^[^ ^33^ ^]^ was used as high‐dimensional abstract descriptors of the local atomic environments that surround each atom in the NPs during the simulations. The SOAP power spectrum had found wide applications in various fields, including materials science, catalysis, and drug discovery. Its ability to encode complex atomic structures and capture subtle variations in local environments makes it a versatile tool for understanding and predicting the behavior of materials at the atomic scale.^[^ ^13^, ^33^, ^41^, ^42^, ^43^ ^]^ In the present work, SOAP spectra of each atom in the NPs were calculated at each of the 1000 MD snapshots taken from the last 1 µ*s* of the simulations (every 1 ns). The *dscribe* ^[^ ^56^ ^]^ was used to generate the SOAP vectors with the following parameters: *r*_*cut*_ = ≈4.48 Å (corresponding to 110%)of the Au FCC lattice parameter, which was included in the calculation up to the first two neighbors in FCC, and up to the third in the HCP case even in case of some small local fluctuations. The *l*_*max*_ parameters for the spherical harmonics were set up to 8, and the *n*_*max*_ parameter was set up to the number of radial basis functions to use to 8. With these parameters, the SOAP spectrum for each atom was a vector of 576 components (of which 324 are unique).

### Atom‐Counting for NPs at High Temperature

The atom counting was based on so‐called scattering cross‐sections (SCSs), representing the total intensity of electrons scattered toward the ADF detector for every atomic column. These SCSs could be quantified using statistical parameter estimation theory.^[^ ^57^, ^58^ ^]^ To achieve this, images were modeled as a superposition of Gaussian functions using the StatSTEM software. From the estimated model parameters, which encompassed the positions, heights, and widths of all atomic columns, the SCS values were determined. In a subsequent analysis, the distribution of the SCSs of all atomic columns was decomposed into overlapping normal distributions, i.e., a Gaussian mixture model. This allowed to count the number of atoms in a particular atomic column with single‐atom sensitivity. However, to ensure the reliability of the results, especially when prior information was lacking, the findings were validated by comparing them to reference SCS values obtained through accurate multislice simulations. These simulations, conducted using MULTEM,^[^ ^59^ ^]^ account for the unique characteristics of the detector, including the non‐uniformity of the real detector surface.^[^ ^60^ ^]^ Furthermore, to achieve the highest level of quantitative accuracy, the temperature‐dependent Debye–Waller factors were incorporated into our image simulations. This accounts for changes in the root mean square deviation of Au atoms concerning temperature variations. An appropriate parameterization^[^ ^61^ ^]^ was used for this purpose.

### Reconstruction Based on Atom‐Counting Results

To obtain the 3D atomic structure of the Au NP from the estimated number of atoms in each atomic column, the proposed method was applied to a simulated system as a validation step. The atom‐counts procedure was used to generate an initial 3D model of the Au NP by arranging the atoms symmetrically around the central plane, based on the known specimen orientation ([110]) and the crystal structure. The distance between adjacent Au atoms was fixed, along the beam direction, according to the lattice parameter.^[^ ^24^ ^]^

Then, an iterative local minima search algorithm was employed to construct the final 3D structure using the starting input model. This process was designed to comprehensively navigate the energy landscape and prevent confinement to nearby local minima. In each iteration, a random atomic column was displaced upward or downward within the interval of [‐a, a], where “a” represents the lattice parameter of the FCC Au structure. Based on the resulting change in the energy of the system, the Boltzmann probability factor (P) was computed,^[^ ^62^, ^63^ ^]^ utilizing Boltzmann's constant (*k*_*B*_) and a selected temperature of 673 K.

(1) $$P=e^{\left(-\Delta E/k_{B}\right)}$$

If P for the candidate structure exceeded a specified threshold, it was accepted and used for the next iteration; otherwise, the previous configuration continued. This process repeated for 2000 iterations, maintaining convexity until deviation due to displaced atomic columns. A threshold of 0.9 was selected for efficient exploration within the energy range near the input model, accounting for computational advantages.

Each candidate structure linked to a local minimum underwent MD relaxation in a canonical ensemble at 673 K for 5 ns using a Nose–Hoover thermostat. Unlike standard energy minimization, this temperature‐specific MD relaxation enabled studying structures observable at elevated temperatures. Indeed, at elevated temperatures, the anisotropy of surface energy diminishes. This phenomenon led to the emergence of rounded features in the equilibrium shape, along with the presence of kinks and steps on the surface. These surface irregularities serve as sites for atom sources and growth, facilitating the diffusion of adatoms.^[^ ^64^ ^]^ During the iterative search and MD simulations, the EAM potential described Au atom interaction, and the interaction between *CeO*_2_ support and the particle was considered using LJ interaction.

To select the most plausible 3D structure, a fitness function (f) was defined.

(2) $$f=\frac{E}{atom}+\alpha \chi$$

It incorporated the average potential energy per atom ($\frac{E}{atom}$) and a quantitative goodness‐of‐fit measure (χ) of candidate structures with the reference observation. The fitness function balanced $\frac{E}{atom}$ and χ using an empirically chosen weighting parameter (α). This function's design was taken from Yu et al.,^[^ ^65^ ^]^ utilizing atom counts and projected atomic column displacements for the discrepancy definition.

The minimum in the fitness graph yielded the final 3D structure aligning best with the reference ADF STEM image in terms of atom count and projected atomic column position.^[^ ^24^ ^]^ The retrieved structure primarily comprises 100 and 111 facets separated by edges and corners. A quantitative comparison with the exact 3D model of the reference image verified the proposed methodology.^[^ ^24^ ^]^ Discrepancies in the number of atoms between reconstructed and original input model atomic columns were minimal, attributed to methodological limitations and atom movement during MD relaxations. Surface structure analysis highlighted an accuracy of over 95% in identifying the reconstructed Au NP's surface structure.^[^ ^24^ ^]^ The iterative local minima search algorithm was then applied to reconstruct the 3D structure of the experimentally investigated NPs of Figure 1g.

It is important to note that in this case, images were recorded sequentially. The recording dwell time per pixel in this ADF STEM experiment was 0.6 µ*s*, and the total recording time required to capture one atomic column is therefore in the range of several microseconds. During that time, the atomic structure was likely to be averaged out experimentally. Each atomic column was revisited after 0.6 s. This sequential recording approach introduces inherent temporal averaging, which could contribute to mitigating the effects of atomic vibrations and enhancing the overall signal‐to‐noise ratio. It effectively accounts for the dynamic nature of atomic motion during imaging, particularly at elevated temperatures. By revisiting each atomic column within a relatively short time frame, the experiment captures a series of atomic snapshots, contributing to a more accurate representation of the dynamic behavior of the NP's atomic structure.

In conclusion, the integration of atom‐counting with an iterative local minima search algorithm, incorporating temperature effects and particle‐support interaction, facilitated the accurate and precise reconstruction of the 3D structures for both simulated and experimentally observed supported NPs. Unlike approaches solely relying on energy minimization, this method outperforms previous techniques that combined 3D atom counting with MC or MD simulations. This approach overcomes inherent limitations by effectively navigating the local energy landscape to pinpoint the local minimum corresponding to the imaged NP structure. This capability enables the successful estimation of target structures, encompassing atomic column positions and surface atomic configuration, as observed in ADF STEM images. Thus, this methodology offers a robust means to retrieve comprehensive 3D atomic‐scale insights into stable and metastable structures within catalytic environments, even at elevated temperatures.

### The Dictionary of SOAP Environments

For the analysis presented here, the well‐defined SOAP distance^[^ ^40^ ^]^ was utilized to classify the environments visited during the simulations, following similar approaches employed in previous studies.^[^ ^41^, ^43^, ^66^ ^]^

The SOAP distance between two SOAP spectra $\vec{a}$ and $\vec{b}$ is calculated as:

(3) $$d_{SOAP}\left(\vec{a},\vec{b}\right)=\sqrt{2-2K(\vec{a},\vec{b})}$$

where, for the SOAP power spectrum representation used in our work, $K\left(\vec{a},\vec{b}\right)=\frac{\vec{a}\cdot \vec{b}}{∥\vec{a}∥∥\vec{b}∥}$

To perform the classification, a dictionary was constructed containing diverse environments from various minimized unsupported Au NPs, aiming to create the most comprehensive dictionary for icosahedral, decahedral, and octahedral NPs' atomic environments. In addition to the NPs simulated in this work, larger‐sized NPs with a higher variety of atomic environments in their ideal state were included to enrich the dictionary.^[^ ^13^ ^]^ The resulting dictionary comprised 47 elements.

To facilitate the usage of the dictionary, its elements were hierarchically classified using the hierarchical clustering algorithms implemented in scipy.^[^ ^67^ ^]^ Initially, the distances between each pair of environments in the dictionary were calculated using Equation (3). Subsequently, a binary tree was created representing the hierarchical classification by employing the “complete” algorithm for hierarchical clustering. This algorithm couples the closest elements at each step and assigns the newly formed couple the largest distance from each remaining element in the set. This process was iteratively applied until the classification was completed.

The resulting binary tree was depicted in the dendrogram shown in Figure 1e. A cut was applied at a distance of 0.08 [*d*_*SOAP*_], resulting in the formation of 10 distinct groups of dictionary entries (as depicted more clearly in Figure 1f) with similar geometrical characteristics, derived from the original 47 environments.

During the analysis of the MD simulations, each environment was assigned to one of these 10 clusters in two steps. First, it was classified as one of the 47 elements in the original environment dictionary by assigning it to the closest element in terms of SOAP distance (using Equation (3)). Then, in the second step, we assigned the analyzed environment to the cluster to which its closest reference belonged.

The dendrogram in Figure 1f displays the final 10 atomic environments (AEs) analyzed. The ‘b AE’ represents all bulk nanoparticle environments, ‘ss’ and ‘ss’ AEs' denote subsurface AEs, with ‘ss’ covering AEs under FCC(111) and FCC(001) faces and edges, and ‘ss’ for non‐standard subsurface AEs under vertices and convex elements. ‘c’ and ‘c’ AEs' encompass concave environments. ‘s’, ‘e’, and ‘e’ AEs cover all surface AEs, where ‘s’ pertains to FCC(111) and FCC(001) faces, ‘e’ and ‘e’ to edge AEs, and ‘v’ and ‘v’ to vertex AEs. This classification is derived from a coarse‐grained SOAP dictionary used for differentiating native and non‐native AEs in simulated nanoparticles.

### Temporal Analysis

To investigate the temporal behavior, transition matrices were calculated based on the cluster information of each atom throughout the simulation.^[^ ^41^, ^48^ ^]^ Transition matrices were constructed by accumulating a table where the elements represent the number of transitions from state i to state j, or from state i to state i, observed at each time step. The probabilities for an atom to transition to a specific atomic environment (or to remain in the same environment) after each time step (with a time increment of δ*t* = 1 ns in our analyses) were further obtained by normalizing each row to 1. In the figures presenting the transition matrices, unobserved transitions were denoted by blank squares. By employing these methodologies, a detailed analysis of the atomic environments and their temporal behavior in the simulated systems was performed. It was noted that the 1 ns timeframe chosen for the analysis was crucial in distinguishing processes occurring on the nanosecond scale or slower. This consideration significantly reduces the likelihood that the exchanges between atomic environments were merely attributable to thermal vibrations. Additionally, the SOAP spectra for each atom in the nanoparticles were calculated based on 1000 molecular dynamics snapshots, taken every 1 ns from the last microsecond of the simulations. This approach, which uses unprocessed atomic positions, was critical for an accurate representation of the dynamics within the nanoparticles.

## Conflict of Interest

The authors declare no conflict of interest.

## Supporting information

Supporting Information

Supplemental Movie 1

## Data Availability

The data that support the findings of this study are openly available in Zenodo at https://doi.org/10.5182/zenodo.10997962.
