# Supplementary material for: Sampling Real‐Time Atomic Dynamics in Metal Nanoparticles by Combining Experiments, Simulations, and Machine Learning
Source: Adv Sci (Weinh). 2024 Apr 24;11(25):2307261. doi: 10.1002/advs.202307261 (PMC11220678; doi:10.1002/advs.202307261)
Supplement: Supplementary file 1 — Supporting Information [file ADVS-11-2307261-s002.pdf]

ADVANCED SCIENCE
Open Access

Supporting Information

for *Adv. Sci.*, DOI 10.1002/advs.202307261

Sampling Real-Time Atomic Dynamics in Metal Nanoparticles by Combining Experiments, Simulations, and Machine Learning

*Matteo Cioni, Massimo Delle Piane, Daniela Polino, Daniele Rapetti, Martina Crippa, Ece Arslan Irmak, Sandra Van Aert, Sara Bals and Giovanni M. Pavan**

SUPPORTING INFORMATION FOR: Sampling real-time atomic dynamics in metal nanoparticles by combining experiments, simulations, and machine learning

Matteo Cioni[1], **Massimo Delle Piane**[1], **Daniela Polino**[2], **Daniele Rapetti**[1], **Martina Crippa**[1], **Ece Arslan Irmak**[3], **Sandra Van Aert**[3], **Sara Bals**[3], **and Giovanni M. Pavan**[1,2,*]

[1]Department of Applied Science and Technology, Politecnico di Torino, Corso Duca degli Abruzzi 24, 10129 Torino, Italy
[2]Department of Innovative Technologies, University of Applied Sciences and Arts of Southern Switzerland, Polo Universitario Lugano, Campus Est, Via la Santa 1, 6962 Lugano-Viganello, Switzerland
[3]EMAT and NANOlab Center of ExcellenceUniversity of AntwerpGroenenborgerlaan 171, 2020 Antwerp, Belgium
[*]corresponding author: Giovanni M. Pavan (giovanni.pavan@polito.it)

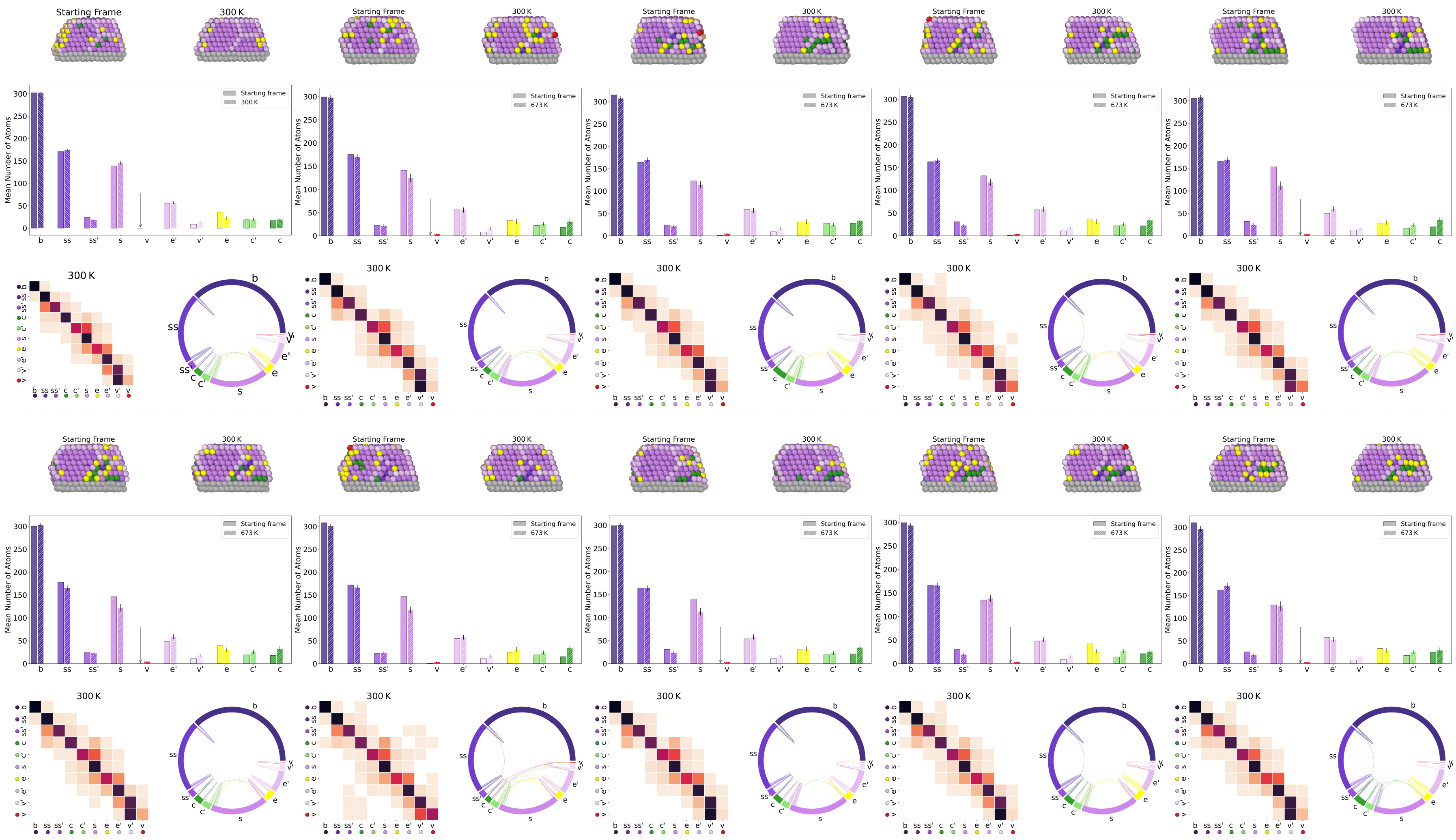

Figure S1. Quantitative analysis of atomic transitions and stability in reconstructed Au NPs at 300 K. (**a**) Initial structures and the corresponding final structures post-MD simulations at both 300K, color-coded according to the SOAP dictionary. (**b**) Histograms representing the average number of atoms per AE during the final microsecond of MD simulations at 300K and 673K. The leftmost column for each AE indicates the initial population, with standard deviations represented by vertical black lines. The absence of certain AEs in the initial structures is denoted by arrows. (**c**) Normalized transition matrices demonstrating the probability of atoms remaining in a given AE (p_{ii}) or transitioning to a different AE ($p_{i \to j}$) within a time interval of $dt = 1$ ns. On right, chord diagrams visualizing the interplay between all AEs within the NP at both temperatures, with the color of the chord corresponding to the AE from which more atoms depart in the pair.

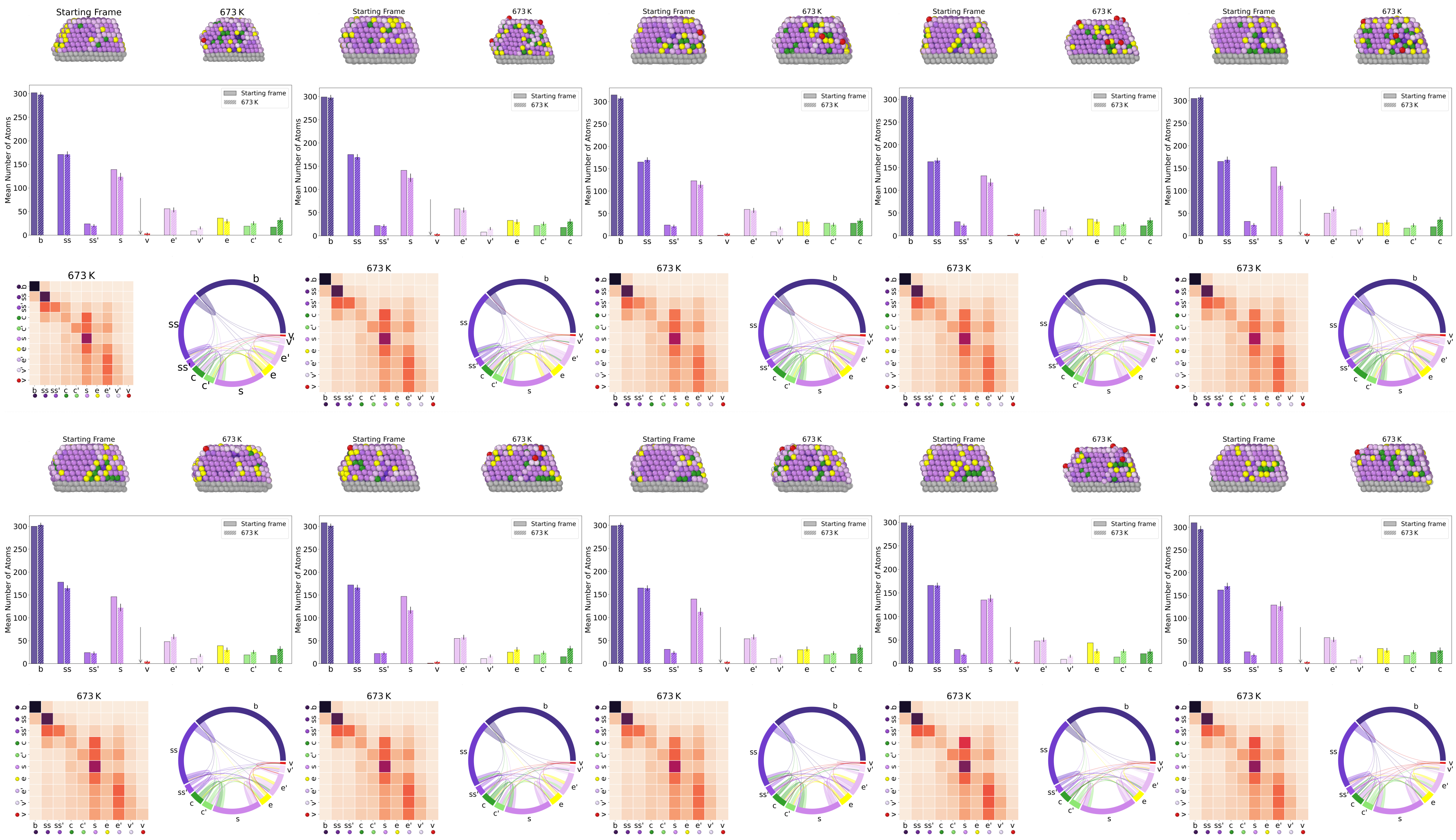

Figure S2. Quantitative analysis of atomic transitions and stability in reconstructed Au NPs at 673 K. (**a**) Initial structures and the corresponding final structures post-MD simulations at both 673K, color-coded according to the SOAP dictionary. (**b**) Histograms representing the average number of atoms per AE during the final microsecond of MD simulations at 300K and 673K. The leftmost column for each AE indicates the initial population, with standard deviations represented by vertical black lines. The absence of certain AEs in the initial structures is denoted by arrows. (**c**) Normalized transition matrices demonstrating the probability of atoms remaining in a given AE (p_{ii}) or transitioning to a different AE ($p_{i \to j}$) within a time interval of $dt = 1$ ns. On right, chord diagrams visualizing the interplay between all AEs within the NP at both temperatures, with the color of the chord corresponding to the AE from which more atoms depart in the pair.
